# Effects of 8-week swimming training on carotid arterial stiffness and hemodynamics in young overweight adults

**DOI:** 10.1186/s12938-016-0274-y

**Published:** 2016-12-28

**Authors:** Wen-Xue Yuan, Hai-Bin Liu, Feng-Shan Gao, Yan-Xia Wang, Kai-Rong Qin

**Affiliations:** 1 Department of Physical Education, Dalian University of Technology, Linggong Road, Dalian, China; 2 Department of Biomedical Engineering, Faculty of Electronic Information and Electrical Engineering, Dalian University of Technology, Linggong Road, Dalian, China; 3 Department of Engineering Mechanics, Dalian University of Technology, Linggong Road, Dalian, China

**Keywords:** Swimming training, Overweight adults, Arterial stiffness, Hemodynamics

## Abstract

**Background:**

Exercise has been found to either reduce or increase arterial stiffness. Land-based exercise modalities have been documented as effective physical therapies to decrease arterial stiffness. However, these land-based exercise modalities may not be suitable for overweight individuals, in terms of risks of joint injury. The purpose of this study was to determine the effects of 8-week swimming training and 4-week detraining on carotid arterial stiffness and hemodynamics in young overweight adults.

**Methods:**

Twenty young male adults who were overweight were recruited and engaged in 8-week of swimming training and 4-week detraining. Five individuals withdrew due to lack of interest and failure to follow the training protocol. Body Fat Percentage (BFP) and carotid hemodynamic variables were measured on a resting day at the following intervals: baseline, 4 weeks, 8 weeks after swimming training and 4 weeks after detraining. A repeated analysis of variance (ANOVA) was used to assess the differences between baseline and each measurement. When significant differences were detected, Tukey’s test for post hoc comparisons was used.

**Results:**

Eight-week swimming training at moderate intensity decreased BFP, including the trunk and four extremities. Additionally, the BFP of the right and left lower extremities continued to decrease in these overweight adults 4 weeks after ceasing training. Carotid arterial stiffness decreased, while there were no significant changes in arterial diameters. Blood flow velocity, flow rate, maximal and mean wall shear stress increased, while systolic blood pressure and peripheral resistance decreased. No significant differences existed in minimal wall shear stress and oscillatory shear stress.

**Conclusions:**

Eight-week swimming training at moderate intensity exhibited beneficial effects on systolic blood pressure, arterial stiffness and blood supply to the brain in overweight adults. Moreover, maximal and mean wall shear stress increased after training. It is worth noting that these changes in hemodynamics did not last 4 weeks. Therefore, further studies are still warranted to clarify the underlying relationship between improvements in arterial stiffness and alterations in wall shear stress.

## Background

Arterial stiffness is an independent risk factor of future cardio- and cerebral events [1]. Common carotid arteries are the main organs that supply blood to the brain. The changes in structure and function of common carotid arteries are relevant with the occurrence and development of atherosclerosis, coronary ischemia and stroke [2]. Local hemodynamics plays an important role in mediating arterial stiffness [3]. Therefore, reducing arterial stiffness via hemodynamic modulation is crucial to the prevention and treatment of cardiovascular disease.

Overweight and obesity are severe public health problems that are common in populations lacking exercises, combined with a hyper caloric intake. Research conducted post-mortem on overweight or obesity suggests that overweight adults usually have severe coronary atherosclerosis, concentric left ventricular hypertrophy, pulmonary embolism, hypoplastic coronary arteries and dilated cardiomyopathies [4]. The metabolic requirements of overweight induce the hemodynamic changes in stroke volume, cardiac output, systolic and diastolic blood pressure as well as alterations in the hypertrophy of smooth muscle arterial walls [5]. Consequently, these changes may accelerate the process of arterial stiffening [6].

Exercise, depending on its modality, has been found to either reduce or increase arterial stiffness [7–12]. Land-based exercise modalities [12], such as walking, running and cycling have been documented as effective physical therapies to decrease arterial stiffness. Unfortunately, these land-based exercise modalities may not be suitable for overweight individuals, in terms of risks of joint injury. Swimming, however, with minimum weight-bearing stress, a humid environment, and a decreased heat load has become an attractive form of exercise and is always recommended for health promotion, and the prevention and treatment of risk factors for cardio-vascular disease [13–16]. Nualnim et al. [16] demonstrated that habitual swimming exercise is an effective endurance exercise for decreasing central arterial stiffness over the age of 50 years. In contrast, Walther et al. [17] suggested that swimmers are more likely to increased arterial stiffness than cyclists. Therefore, additional investigations on the effects of swimming training on arterial stiffness in overweight or obese individuals are needed.

A number of investigations [18, 19] have shown that hemodynamic variables including blood pressure, blood-flow-induced wall shear stress (WSS), and oscillatory shear index (OSI) play vital roles in modulating arterial stiffness. Exercise can directly alter systemic and local hemodynamic variables [7]. Vascular endothelial and smooth muscle cells in the blood vessels may sense these hemodynamic responses, resulting in cellular responses, such as changes in cell morphology, cell function, and gene expression, which are more relevant with changes in arterial stiffness [20]. To date, most studies [9, 21] have focused on the effects of acute exercise on arterial stiffness and hemodynamics, without full consideration of the alteration in hemodynamic responses to long term exercise. Lawrence [4] manifested hemodynamic changes (heart rate, systolic and diastolic blood pressure) in overweight and obese individuals, following 8 weeks of home-based calisthenics training. Recently, Shaw [22] reported the effects of 8 weeks concurrent resistance and aerobic training on hemodynamics (resting heart rate, systolic, diastolic and mean blood pressure) in overweight and obese populations. Despite substantial progress, relatively little information is available concerning the effects of swimming training on arterial stiffness and hemodynamics (blood pressure, peripheral resistance, wall shear stress, and oscillatory shear index) in overweight individuals.

The purpose of this study was to explore the effects of swimming training on carotid arterial stiffness and hemodynamics in overweight adults. The study used 8 weeks of supervised swimming training and a further 4 weeks of ceased training to assess the outcomes of training and detraining.

## Methods

### Subjects

Twenty male volunteers, aged from 19 to 21, were recruited from the surrounding districts of the university in this study. The subjects had no history of cardiovascular disease or any other medical disorder were overweight (body mass index (BMI), 30 ± 3 kgm^−2^) and were not involved in any regular, planned exercise program [23] during the past 3 months. Subjects were required to have swimming skills including crawl, breaststroke or both. None of the subjects had taken cardiovascular or blood pressure medicines. During the swimming intervention, three individuals withdrew, due to lack of interest in the study. A further two individuals withdrew from the detraining, due to not ceasing swimming activity. The present study was approved by the Ethics Committee, Dalian University of Technology, China. All subjects provided written informed consent before inclusion.

### Experimental design

**Fig. 1:**
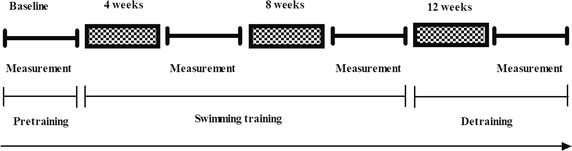
Protocol of swimming training and study methodology

Subjects visited the lab four times during the supervised swimming training (Fig. 1), and each subject’s visit was performed at the same time. At the intervals of baseline, 4 and 8 weeks after swimming training and 4 weeks of detraining, body fat percentage and hemodynamics were measured on a resting day.

### Swimming training protocol

Swimming training was organized at an indoor swimming pool with mean water temperature of 25.5 °C. Subjects completed supervised training three times per week for 8 weeks. Each training session consisted of 5 min stretching on land, a 5 min kicking exercise in the water, 30 min swimming, a 10 min cool down, and 5 min stretching. Swimming, including front crawl and breaststroke, was performed as interval training with rest times declining, as fitness improved. In the first 2 weeks, subjects swimming, exercised at 50% maximal heart rate (HR_max_), and exercised at 65–80% HR_max_ intensity from 3 weeks to 8 weeks. HR was accessed by heart rate monitor (Geonaute 8360801, France).

### Body fat percentage and hemodynamics measurement

#### Body fat percentage (BFP) measurement

Body fat percentage was measured by bioelectrical impedance (model TBF-418B, Tanita Corp, Japan). Subjects wore light clothing and no shoes. During the measurements, the subjects stood erect with feet shoulder-width apart.

#### Hemodynamics measurement

The inner arterial diameters and blood flow velocity waveforms measurements were examined using a high-resolution Doppler ultrasound (ProSound Alpha 7, Aloka). The heart rate, brachial systolic pressure (*p*_*s_mea*_), and diastolic pressure (*p*_*d_mea*_) were simultaneously assessed on the left upper arm with a cuff-type manometer (Patient Monitor PM8000, Mindray) and repeated in triplicate, and the average of the three values was calculated.

### Calculation of hemodynamic variables

#### Blood pressure (BP)

In this study, the mean value of the carotid arterial pressure *p*_*m*_ and diastolic pressure *p*_*d*_ were assumed to be equal to the mean value of the brachial pressure *p*_*m_mea*_ and diastolic pressure *p*_*d_mea*_, as performed in a previous investigation [10]. The mean arterial pressure (*p*_*m*_) was calculated using the following equation:

1 $$p_{m} = p_{m\_mea} = p_{d\_mea} + \frac{1}{3}\left( {p_{s\_mea} - p_{d\_mea} } \right)$$

Therefore, the carotid artery blood pressure waveform was calibrated using the brachial mean arterial *p*_*m_mea*_ and diastolic pressure *p*_*d_mea*_. The maximal value of the carotid arterial pressure waveform was then calculated and assumed to be the systolic pressure *p*_*s*_.

#### Flow rate (FR)

The FR was computed as

2 $$Q = 2\pi R_{0}^{2} \int_{0}^{1} {y \cdot u(y) \cdot dy} ,$$

where *R*_0_ is the time-averaged value of the carotid artery radius in one cardiac cycle, *y* = *r*/*R*_0_ in which *r* is the radial coordinate, and *u*(*y*) satisfies [24]

3 $$u(y,t) = \sum\limits_{n = - \infty }^{ + \infty } {\frac{{J_{0} (\alpha_{n} j^{{\frac{3}{2}}} ) - J_{0} (\alpha_{n} j^{{\frac{3}{2}}} y)}}{{J_{0} (\alpha_{n} j^{{\frac{3}{2}}} ) - 1}}u(0,\omega_{n} )e^{{j\omega_{n} t}} } ,$$

where *n* is the harmonic number, *J*_*0*_ is the 0th-order Bessel function of the first kind, and $j = \sqrt { - 1}$, $\alpha_{n} = R_{0} \sqrt {{{\rho \omega_{n} } \mathord{\left/ {\vphantom {{\rho \omega_{n} } \eta }} \right. \kern-0pt} \eta }}$ is the Womersley number. *ρ* is the density of blood, *η* is blood viscosity. *η* and *ρ*, in the present study, were taken as the same values for all subjects, i.e., *η* = 0.004 Pa·s and *ρ* = 1050 kg/m^3^, respectively. *ω*_*n*_ = 2*nπf* is the angular frequency, and *f* is the base frequency. *u*(0, *ω*_*n*_) is the *n* harmonic component of the measured center-line velocities. The maximal harmonic number *n* was computed as 20 and satisfies

4 $${\text{u}}(0,t) = \sum\limits_{n = - \infty }^{ + \infty } {u\left( { 0 ,\omega_{n} } \right)e^{{j\omega_{n} t}} } .$$

*V*_*max*_ *, V*_*min*_, and *V*_*mean*_ are the maximal, minimal, and mean center-line velocities, after one cardiac cycle. *Q*_*max*_, *Q*_*min*_, and *Q*_*mean*_ are the maximal, minimal, and mean blood flow FR, after one cardiac cycle.

#### *β*-stiffness index (*β*)

*β* was calculated as a means of adjusting arterial compliance for changes in distending pressure as follows [8]:

5 $$\beta = \frac{{{ \ln }\left( {{{{\text{p}}_{\text{s}} } \mathord{\left/ {\vphantom {{{\text{p}}_{\text{s}} } {{\text{p}}_{\text{d}} }}} \right. \kern-0pt} {{\text{p}}_{\text{d}} }}} \right)}}{{{\text{R}}_{\text{s}} - R_{d} }} \cdot R_{d} .$$

#### Peripheral resistance (R_P_)

6 $$R_{p} = \frac{{p_{mean} }}{{Q_{mean} }}$$

#### Wall shear stress (WSS)

The blood flowing along the vascular vessel creates a tangential friction force, known as wall shear stress (*τ*_*w*_), and was computed as [24]:

7 $$\tau_{w} = \frac{\eta }{{R_{0} }}\sum\limits_{n = - \infty }^{ + \infty } {\frac{{\alpha_{n} j^{{\frac{3}{2}}} J_{1} (\alpha_{n} j^{{\frac{3}{2}}} )}}{{J_{0} (\alpha_{n} j^{{\frac{3}{2}}} ) - 1}}u(0,\omega_{n} )e^{{j\omega_{n} t}} } ,$$

where *J*_*1*_ is the first-order Bessel function of the first kind. *τ*_*w_max*_, *τ*_*w_min*_, and *τ*_*w_mean*_ refer to the maximal, minimal, and mean shear stress waveforms, after a cardiac cycle.

#### Oscillatory shear index (OSI)

The OSI is an index that describes the shear stress acting in directions other than the direction of the temporal mean shear stress vector and was defined by Ku et al. [25] as

8 $${\text{ OSI = }}\frac{1}{2}\left( { 1{ - }\frac{{\left| {\int_{ 0}^{T} {\tau_{\text{w}} dt} } \right|}}{{\int_{0}^{T} {\left| {\tau_{\text{w}} } \right|} dt}}} \right)$$

where, *T* is the period of one cardiac cycle.

### Statistical analysis

For data management and analysis, SPSS 20.0 software (SPSS Inc., Chicago, IL, USA) was used. All values were presented as the mean ± SD. The repeated ANOVA was used to assess differences between baseline and each measurement. When significant differences were detected, Tukey’s test was used for post hoc comparisons. The significance level was set at *P* = 0.05.

## Results

### Effects on body fat percentage

The changes in body fat percentage of subjects during 8-week training and 4-week detraining are presented in Table 1. There were significant differences between baseline and after 8-week training in the fat percentages of the whole body, trunk, left UE, left LE, right UE, and right LE. Compared with baseline, after 4 weeks detraining, there were significant differences in the fat percentages of the left LE, and right LE.

**Table 1:** Effects of swimming training on the body fat percentage

Fat percentage	Pretraining	Swimming training		Detraining
	Baseline	4 weeks	8 weeks	12 weeks
Whole body	28.6 ± 5.7	27.3 ± 6.1	25.4 ± 5.6*	25.8 ± 6.1
Trunk	29.8 ± 5.8	28.8 ± 5.6	26.1 ± 6.0*	27.1 ± 5.2
Left UE	25.9 ± 6.4	24.6 ± 6.2	23.9 ± 5.1*	24.6 ± 6.3
Left LE	27.8 ± 6.1	27.2 ± 5.9	25.2 ± 6.1*	25.5 ± 5.8*
Right UE	25.3 ± 6.2	24.8 ± 5.6	23.2 ± 5.0*	23.9 ± 6.0
Right LE	27.9 ± 6.2	27.3 ± 5.7	25.1 ± 6.2*	26.1 ± 5.8*

*UE* upper extremity, *LE* lower extremity, Unit: %

* Significant difference from baseline: *P* < 0.05

### Effects on arterial stiffness and diameters

**Fig. 2:**
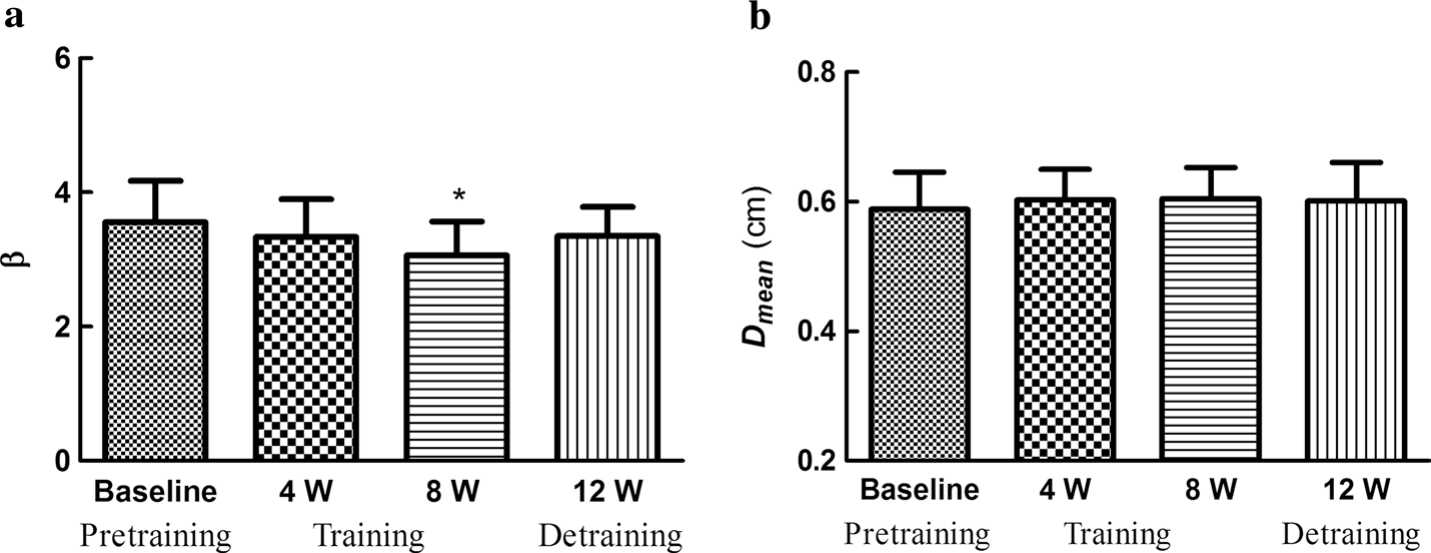
Effects on arterial stiffness and mean arterial diameters. ** a** Arterial stifness (*β*).** b** Mean arterial diameters (*D*_*mean*_)

Figure 2 shows the changes in arterial stiffness and diameters before and after swimming training. Compared to baseline, carotid arterial stiffness was significantly lower at 8 weeks after training. There were no significant differences in mean arterial diameters between baseline, post training and detraining.

### Effects on blood flow velocity and blood flow rate to brain

**Fig. 3:**
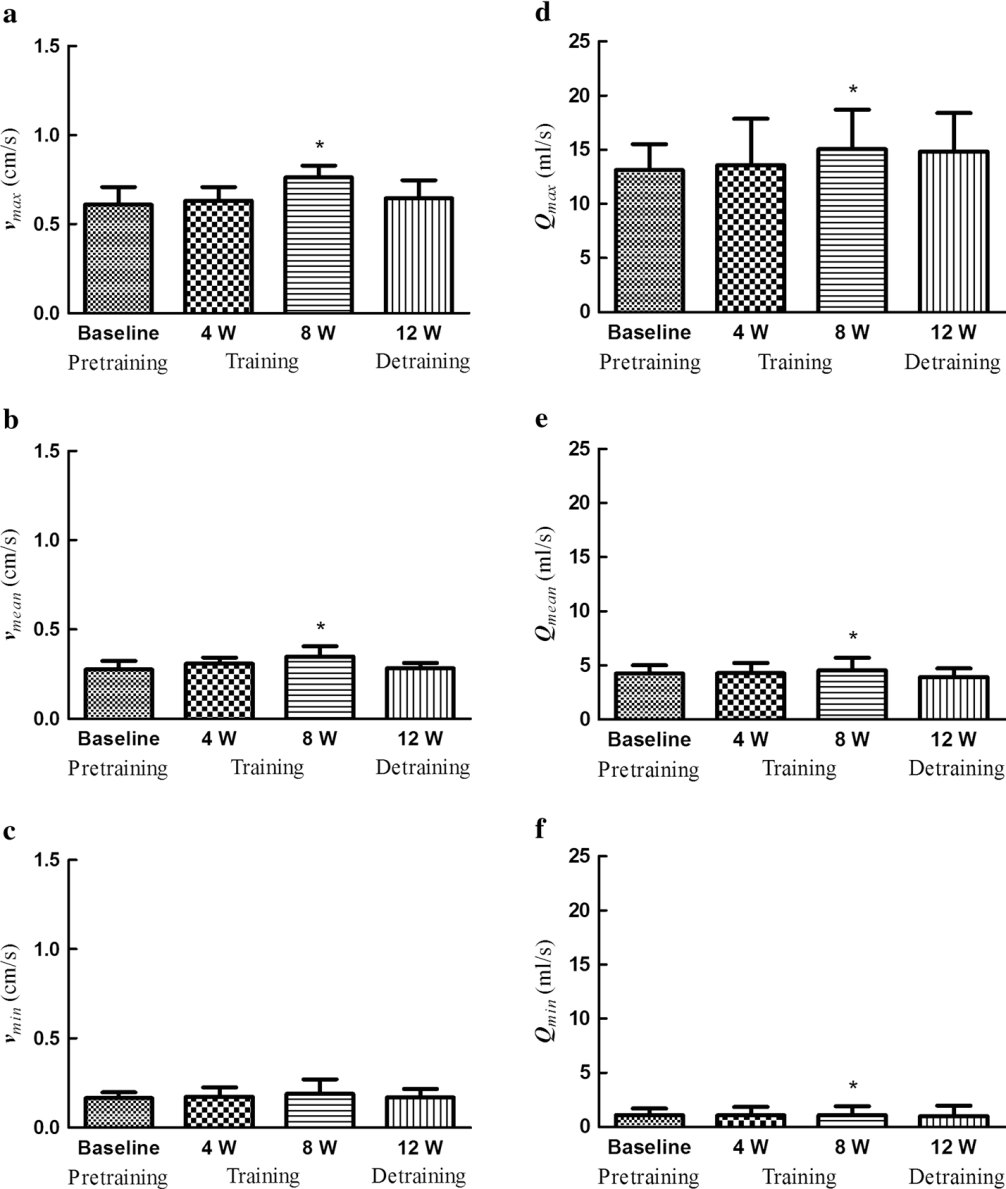
Effects on blood flow to brain.** a** Maximal center-line velocity (*v*_*max*_).** b** Mean center-line velocity (*v*_*mean*_).** c** Minimal center-line velocity (*v*_*min*_).** d** Maximal flow rate (* Q*_*max*_). ** e** Mean flow rate (* Q*_*mean*_). ** f** Minimal flow rate (*Q*_*min*_)

Figure 3a, b and c illustrate that maximal, mean center line velocities were significantly increased after 8-week swimming training. Figure 3d, e and f display, compared with baseline, the maximal, mean and minimal flow rates, which were significantly increased after 8-week swimming training.

### Effects on blood pressure, peripheral resistance, wall shear stress and OSI

**Fig. 4:**
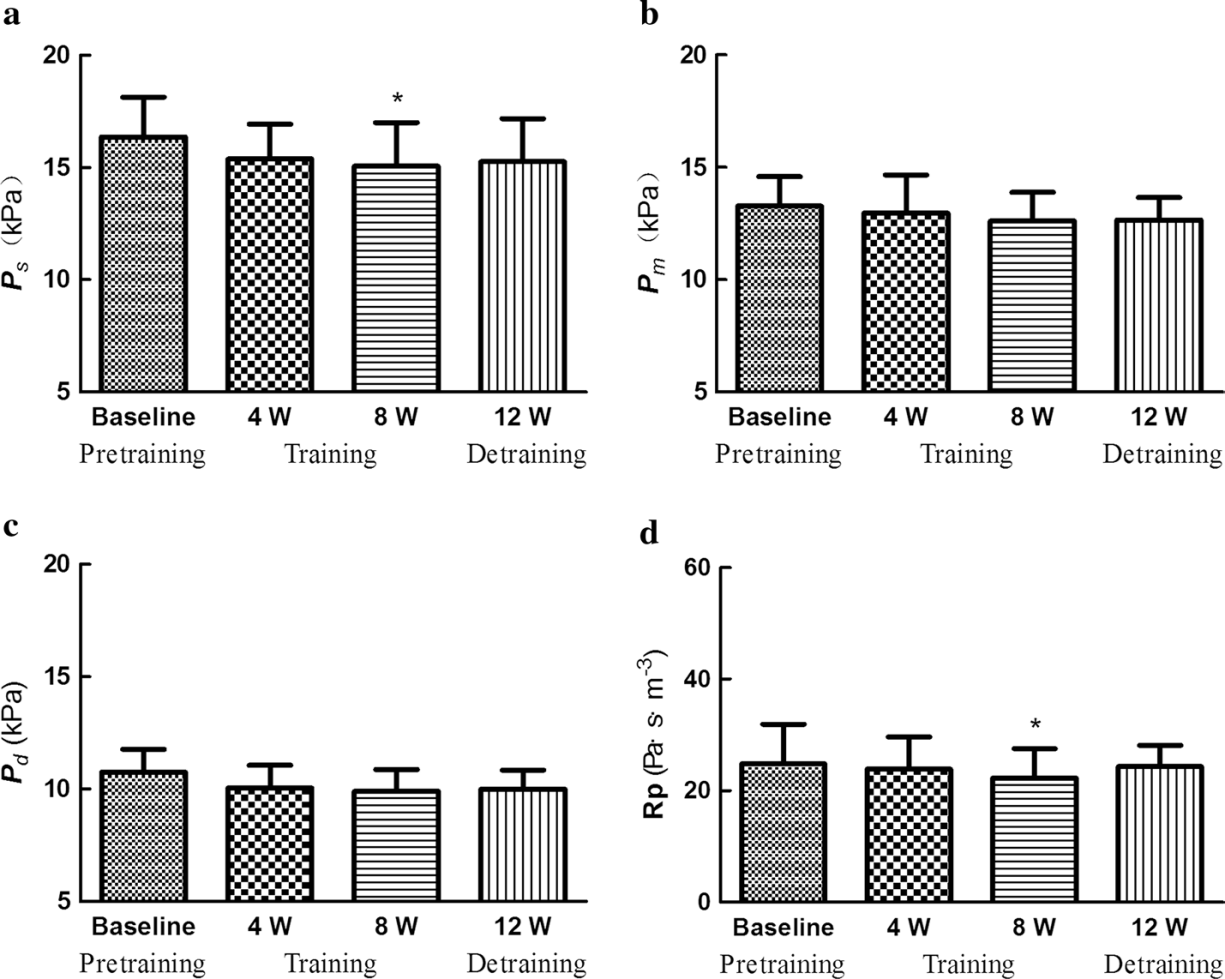
Effects on blood pressure and peripheral resistance. ** a** Systolic blood pressure (***P***_*s*_).** b** Mean blood pressure (***P***_*m*_).** c** Diastolic blood pressure (***P***_*d*_).** d** Peripheral resistance (**Rp**)

**Fig. 5:**
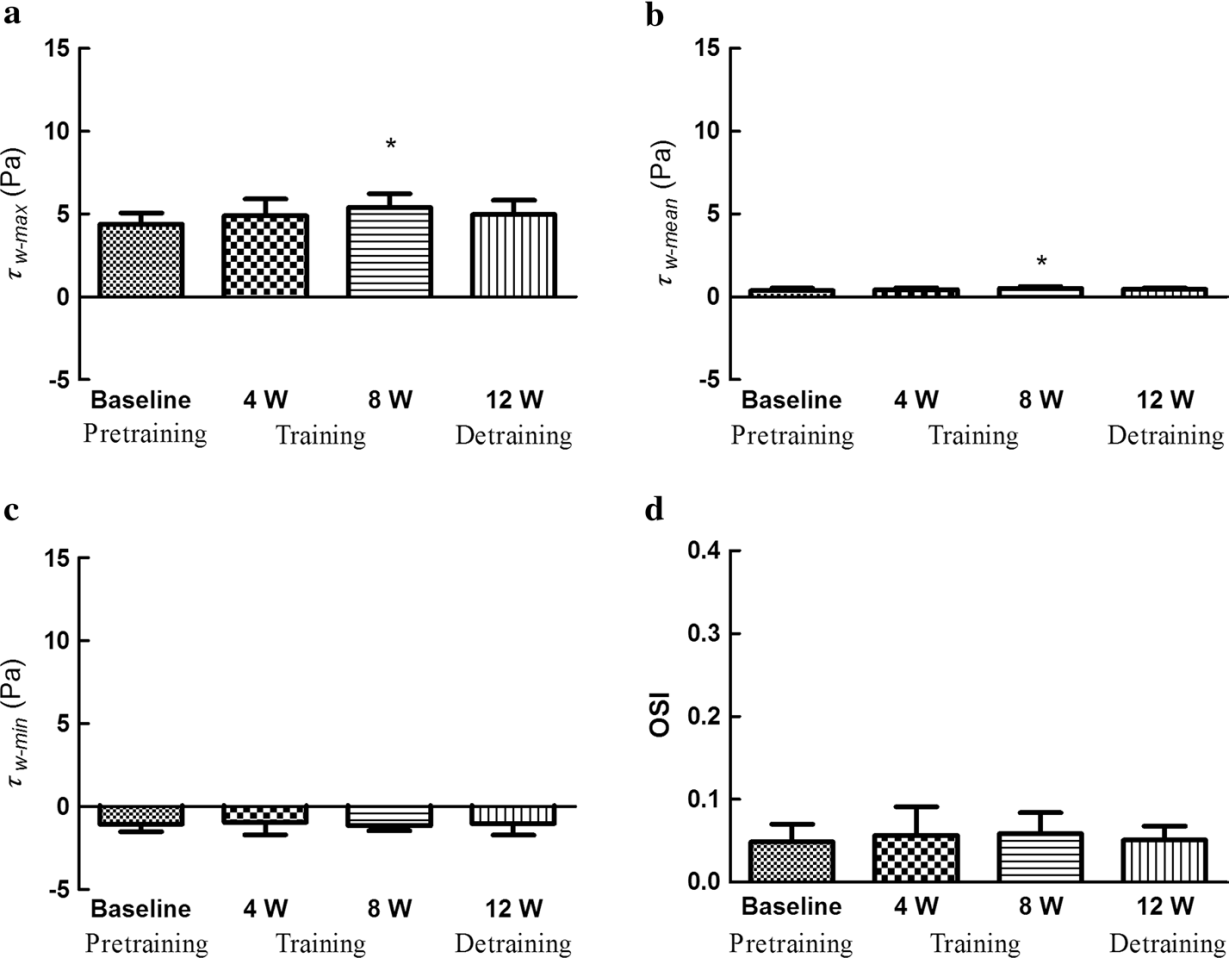
Effects on wall shear stress (WSS) and OSI.** a** Maximal wall shear stress (*τ*_*w−max*)_. ** b** Mean wall shear stress (*τ*_*w−mean*)_. **c** Minimal wall shear stress (*τ*_*w−min*)_. **d** Oscillatory shear index (OSI)

Figure 4 shows that compared with baseline, both systolic blood pressure and peripheral resistance decreased after 8-week swimming training. Figure 5 illustrates that maximal and mean wall shear stress increased after 8-week training, while no significant difference existed in oscillatory shear stress.

## Discussion

Swimming is always recommended for overweight individuals to improve cardiovascular function [13–16]. However, research on the effects of swimming exercise on arterial stiffness are extremely limited and controversial [16, 17]. Additionally, the hemodynamic variables, induced by exercise, play vital roles in modulating arterial stiffness [3, 18, 19]. Some studies have reported the effects of home-based training on hemodynamic changes in overweight individuals [4, 22]. To date, little information is available concerning the effects of swimming training on arterial stiffness and hemodynamics in overweight individuals. The purpose of this study was to detect the effects of swimming training on carotid arterial stiffness and hemodynamics in overweight adults. The main results of 8 weeks of moderate intensity swimming training can be summarized as follows: (1) Carotid arterial stiffness was decreased while arterial diameters were not changed. (2) Blood supply to the brain via carotid arteries was improved. (3) All hemodynamic variables, including blood pressure, peripheral resistance, wall shear stress and OSI in this study were significantly changed but returned to baseline levels by 4 weeks after detraining.

The decreases in body fat percentage with swimming have important implications for overweight individuals in this study. Previous studies [26] have suggested that water-based exercise may be less effective than land-based modalities, such as walking or running, because of different effects on energy balance and weight loss mechanisms. The findings in this paper indicate that 8 weeks of swimming training, at moderate intensity, reduced whole body fat percentage and had a continuous effect on the lower extremities.

Swimming training is perceived as an excellent form of exercise, especially for the overweight who suffers from increased risk of cardiovascular disease, because swimming involves minimum weight-bearing stress [13]. However, research on the effects of swimming exercise, on cardiovascular health profile is extremely limited. The results in this paper demonstrated that 8 weeks of swimming training at moderate intensity can significantly increase blood velocity and flow rate, and decrease systolic BP and peripheral resistance. Tanaka et al. [27] presented the results of 10 weeks of a swimming program, demonstrating a decrease in systolic and diastolic blood pressure. Recently, Alkatan’s group [13] reported that systolic BP was reduced after both 12 weeks of swimming and cycling training. The results in this study confirm, and extend the above findings by demonstrating that 8 weeks of swimming training at moderate intensity decreased systolic BP, instead of mean and diastolic BP.

Several cross-sectional and longitudinal studies have indicated that exercise training is correlated with enlargement of the large arteries (aorta, carotid and femoral artery) in humans. Endurance-trained athletes were reported to possess larger arteries than control individuals [10, 28, 29]. Additionally, the resting femoral artery diameter was enhanced after walking training in sedentary men [30]. No significant increase in carotid arterial diameter was detected in this study. Conversely, it is well-documented that regular land-based exercise training, such as running and cycling, can reduce arterial stiffness [12], even if the changes in arterial stiffness are obtained after 1 week of aerobic running exercise [31]. The arterial stiffness in this study decreased significantly after 8 weeks of swimming training. This finding is consistent with the notion [15] that regular swimming exercise plays an important role in preventing arterial stiffening. The above-mentioned contrary conclusions [15–17] may be drawn from different subjects, exercise intensity, or different intervention protocols.

One potential explanation for the vascular adaptations to exercise training relates to shear stress [32]. Endothelial cells (ECs) along blood vessels can sense variations of WSS and contribute to the endothelial production of vasoactive mediators, such as nitric oxide, which can cause changes in arterial function and structure [20]. It is well established that low wall shear stress (WSS) may be involved in the early stages of the atherosclerotic process [33, 34]. Gnasso et al. [35] proposed that WSS in the common carotid artery is inversely associated with intima-media thickness, age, systolic BP and BMI in healthy male subjects. More recent investigations [36, 37] suggested that the mean WSS significantly decreases in both sexes with age, while peak WSS decreases significantly only in men. Despite the fact that hemodynamic shear stress is a major determinant of vessel diameter and vascular remodeling [18, 19], little information is available regarding the influence of regular swimming on WSS. The results in this study suggest that maximal and mean WSS significantly increased after 8 weeks of swimming training. According to the formula [24], WSS is determined by flow velocity and whole blood viscosity and is inversely related to vessel diameter [33]. The data in this study are limited by the fact that blood viscosity was at the same value for all subjects. The changes in arterial structure and function may relate to the impact of swimming training on blood flow and shear stress patterns. Therefore, the impact of increases in maximal and mean WSS at carotid artery, in relation to the changes in structure and function, remain to be established.

The investigations of home-based training on the hemodynamic variables in overweight and obese populations are limited only to heart rate, and systolic and diastolic blood pressure [4, 22]. In this study, hemodynamics, including blood pressure, peripheral resistance, wall shear stress, and oscillatory shear index were measured and computed to examine the effects of swimming training on carotid arterial stiffness and hemodynamics. This study not only presents hemodynamic information for identifying an effective form of exercise for improving arterial stiffness but also serves as a basis for a further understanding of the hemodynamic mechanisms underlying the modulation of arterial stiffness via exercise training.

## Conclusions

In this study, 8-week swimming training at moderate intensity exhibited beneficial effects on systolic blood pressure, arterial stiffness and blood supply to the brain in overweight adults. Additionally, maximal and mean WSS were increased after 8 weeks training. It is worth noting that these changes in hemodynamics did not last 4 weeks. Therefore, further studies are still needed to clarify the underlying relationship between improvements in arterial stiffness and alterations in WSS.
